# Supplementary material for: Modeling glioblastoma heterogeneity as a dynamic network of cell states
Source: Mol Syst Biol. 2021 Sep 16;17(9):e10105. doi: 10.15252/msb.202010105 (PMC8444284; doi:10.15252/msb.202010105)
Supplement: Supplementary file 5 — Source Data for Figure 3 [file MSB-17-e10105-s001.zip › Figure3A_sourcedata/GSEA_3065/nosigdb_state3.GseaPreranked.1622718426028/DOROSZKO_U3013MG_CLUSTER_0.html]

Details for gene set DOROSZKO\_U3013MG\_CLUSTER\_0[GSEA]

|  || Dataset | state3 |
| Phenotype | NoPhenotypeAvailable |
| Upregulated in class | na\_pos |
| GeneSet | DOROSZKO\_U3013MG\_CLUSTER\_0 |
| Enrichment Score (ES) | 0.6262268 |
| Normalized Enrichment Score (NES) | 1.8712342 |
| Nominal p-value | 0.0 |
| FDR q-value | 0.002570413 |
| FWER p-Value | 0.025 |
Table: GSEA Results Summary

  

Fig 1: Enrichment plot: DOROSZKO\_U3013MG\_CLUSTER\_0      
 Profile of the Running ES Score & Positions of GeneSet Members on the Rank Ordered List

  

| PROBE | GENE SYMBOL | GENE\_TITLE | RANK IN GENE LIST | RANK METRIC SCORE | RUNNING ES | CORE ENRICHMENT || 1 | PTPRZ1 |  |  | 14 | 0.532 | 0.1045 | Yes |
| 2 | SYT11 |  |  | 111 | 0.335 | 0.1603 | Yes |
| 3 | NES |  |  | 113 | 0.332 | 0.2264 | Yes |
| 4 | BCHE |  |  | 136 | 0.315 | 0.2866 | Yes |
| 5 | IGFBP2 |  |  | 164 | 0.303 | 0.3440 | Yes |
| 6 | TNC |  |  | 249 | 0.264 | 0.3872 | Yes |
| 7 | COL6A1 |  |  | 254 | 0.261 | 0.4387 | Yes |
| 8 | MEG3 |  |  | 258 | 0.260 | 0.4903 | Yes |
| 9 | NOVA1 |  |  | 270 | 0.253 | 0.5395 | Yes |
| 10 | VCAN |  |  | 293 | 0.245 | 0.5859 | Yes |
| 11 | MAP2 |  |  | 344 | 0.231 | 0.6262 | Yes |
| 12 | MAP1B |  |  | 975 | 0.140 | 0.5820 | No |
| 13 | PCSK1N |  |  | 1484 | 0.104 | 0.5446 | No |
| 14 | MEF2C |  |  | 2482 | 0.058 | 0.4418 | No |
| 15 | IGFBP5 |  |  | 3555 | 0.027 | 0.3242 | No |
| 16 | PGM2L1 |  |  | 3831 | 0.019 | 0.2966 | No |
| 17 | GSN |  |  | 4064 | 0.013 | 0.2727 | No |
| 18 | SNX22 |  |  | 4498 | 0.001 | 0.2233 | No |
| 19 | DMD |  |  | 5320 | -0.019 | 0.1330 | No |
| 20 | NGFR |  |  | 5771 | -0.032 | 0.0878 | No |
| 21 | SNX1 |  |  | 5937 | -0.037 | 0.0763 | No |
| 22 | ANXA1 |  |  | 7032 | -0.083 | -0.0325 | No |
| 23 | CKB |  |  | 7659 | -0.134 | -0.0775 | No |
| 24 | ANXA2 |  |  | 8206 | -0.230 | -0.0942 | No |
| 25 | CAVIN1 |  |  | 8208 | -0.231 | -0.0483 | No |
| 26 | CAMK2N1 |  |  | 8713 | -0.554 | 0.0044 | No |
Table: GSEA details [plain text format]

  

Fig 2: DOROSZKO\_U3013MG\_CLUSTER\_0: Random ES distribution      
 Gene set null distribution of ES for **DOROSZKO\_U3013MG\_CLUSTER\_0**
